# Vitamin E

**DOI:** 10.3390/antiox7030044

**Published:** 2018-03-20

**Authors:** Volker Böhm

**Affiliations:** Institute of Nutrition, Friedrich Schiller University Jena, Dornburger Str. 25-29, 07743 Jena, Germany; Volker.Boehm@uni-jena.de

Vitamin E is the major lipid-soluble antioxidant in the cell antioxidant system and is exclusively obtained from the diet. Vitamin E protects polyunsaturated fatty acids and other components of cell membranes and low-density lipoproteins from oxidation by free radicals. It is located primarily within the phospholipid bilayer of cell membranes. The most important form is α-tocopherol. Clinical signs of deficiency occur very rarely [1].

During the last ca. 100 years since the discovery of vitamin E, research has focused on different properties of this molecule, the focus often depending on the specific techniques and scientific knowledge present at each time. Originally discovered as a dietary factor essential for reproduction in rats, vitamin E has revealed many more important molecular properties meanwhile, such as the scavenging of reactive oxygen and nitrogen species with consequent prevention of oxidative damage associated with many diseases, or the modulation of signal transduction and gene expression in antioxidant and non-antioxidant manners [2].

This special issue highlights some of the recent advances in vitamin E research, showing the status quo on the one hand and providing new insights in functions and physiological relevance on the other hand. The review of Mène-Saffrané summarizes the current knowledge of tocochromanol biosynthesis in plants and highlights future challenges regarding the understanding of its regulation [3]. Adding to this topic, Fritsche et al. [4] review 30 years of research on tocopherols in model and crop species, with special emphasis on the improvement of vitamin E content using transgenic approaches and classical breeding [4]. Kodad et al. [5] report results for one of the most important nut species worldwide, the almond. Almond kernels show the highest levels of tocopherols among all nuts, being dependent on the genotype and the environment [5].

Reboul [6] describes the fate of vitamin E in the human gastrointestinal lumen during digestion. She focuses on the proteins involved in the intestinal membrane and cellular transport of vitamin E across the enterocyte and discusses factors modulating vitamin E micellarization and absorption. During the metabolism of vitamin E, the long-chain metabolites 13’-hydroxychromanol and 13’-carboxychromanol are formed by oxidative modification of the side-chain [7]. Their occurrence in human serum indicates a physiological relevance. Effects of these metabolites on lipid metabolism, apoptosis, proliferation, and inflammatory actions have been shown. Interestingly, the long-chain metabolites exerted effects different from that of their precursors [7]. Comitato et al. [8] review special biological activities of tocotrienols, not shared by tocopherols. Thus, tocotrienols showed the ability to inhibit cancer cell growth and induce apoptosis thanks to specific mechanisms. In addition, neuroprotective activities of tocotrienols are also presented [8].

Another part of this special issue presents specific effects of vitamin E. Mutalip et al. [9] answers the question: What are the known roles of vitamin E as an antioxidant in female reproductive health? This paper comes back to the initial discovery of vitamin E in 1922 as a substance necessary for reproduction. El Hadi et al. [10] discuss vitamin E as a potent antioxidant being able to reduce oxidative stress in nonalcoholic fatty liver disease. They also present therapeutic efficacy. Nukala et al. [11] discuss another interesting application. They describe the efficacy of tocopherols and tocotrienols as radiation countermeasures and identify the challenges to be addressed to develop them into radiation countermeasures for human use. Mohn et al. [12] describe the membrane distribution of α-tocopherol in brain regions of adult rhesus monkeys. These authors also look for associations between membrane α-tocopherol and content of polyunsaturated fatty acids.
